# Artificial intelligence–enabled nursing surveillance for pediatric cancer immunotherapy: integrating biomarkers, patient-reported outcomes, and long-term toxicity management

**DOI:** 10.3389/fmed.2026.1976674

**Published:** 2026-10-16

**Authors:** Yi Peng, Qiong Kou, Sihong Guo, Jia Xu, Lijuan Wang, Xiaoyan Chen

**Affiliations:** 1 Department of Pediatrics, The First People’s Hospital of Guangyuan, Guangyuan, Sichuan, China; 2 Department of Gynecology, The First People’s Hospital of Guangyuan, Guangyuan, Sichuan, China

**Keywords:** artificial intelligence, cancer immunotherapy, cytokine release syndrome, long-term toxicity, nursing surveillance, patient-reported outcomes, pediatric oncology, survivorship

## Abstract

Pediatric cancer immunotherapy has expanded from anti-GD2 antibodies and bispecific T-cell engagers to immune checkpoint inhibitors and chimeric antigen receptor T-cell therapy. These treatments can produce rapid, nonlinear, and partially overlapping toxicities, including cytokine release syndrome, immune effector cell-associated neurotoxicity syndrome, infection, prolonged cytopenia, B-cell aplasia, and organ-specific immune-related adverse events. Nurses provide the highest-frequency clinical observations across inpatient, ambulatory, and home settings, yet conventional monitoring often fragments laboratory data, vital signs, neurologic findings, caregiver observations, and patient-reported outcomes. Artificial intelligence (AI) offers a means to fuse these longitudinal signals into individualized risk trajectories. The clinically relevant mechanism, however, is not autonomous prediction; it is a nurse-in-the-loop system in which AI improves temporal sensitivity and prioritization, while nurses verify signal quality, interpret age- and development-specific context, reconcile child–caregiver discordance, activate protocol-based escalation, and maintain accountability. This Mini Review examines how biomarkers, continuous physiological monitoring, electronic patient-reported outcomes, and caregiver-reported behavioral changes can support AI-enabled nursing surveillance during acute immunotoxicity and long-term survivorship. We propose a three-layer framework linking immune-toxicity biology, a multimodal digital phenotype, and nursing counter-regulation. We also discuss workflow integration, alert fatigue, model drift, bias, privacy, and the need for prospective pediatric validation. AI should augment not replace specialist pediatric oncology nursing judgment and should be evaluated by whether it improves timely intervention, symptom burden, family experience, and long-term safety.

## 1 Introduction

Survival has improved for many childhood cancers, but relapsed and refractory disease remains a major clinical problem, particularly for high-risk leukemia and solid tumors (1). Immunotherapies now occupy an established or emerging role across pediatric oncology. Tisagenlecleucel can induce durable remission in relapsed or refractory B-cell acute lymphoblastic leukemia, blinatumomab has improved outcomes in selected relapsed B-ALL settings, anti-GD2 immunotherapy is integral to high-risk neuroblastoma care, and GD2-directed CAR-T cells have shown activity in refractory neuroblastoma (2–5). Checkpoint inhibitors have demonstrated meaningful activity in a limited subset of pediatric tumors, especially Hodgkin lymphoma and tumors with selected molecular features, while overall response rates in unselected pediatric solid tumors remain modest (6, 7). Emerging cell-based platforms, including CAR-NK therapy, further broaden the immunotherapy landscape, although pediatric clinical experience remains limited (8, 9). Recent work also illustrates the growing use of AI to interrogate tumor immune microenvironments and evolving immune-checkpoint biology (10, 11).

The monitoring burden created by these therapies differs from that of conventional chemotherapy. Immune-mediated toxicities can evolve over hours, present with nonspecific symptoms, recur after apparent stabilization, or emerge months after treatment. Fever may represent infection, cytokine release syndrome (CRS), or both; somnolence may reflect medication, sleep disruption, metabolic disturbance, or immune effector cell-associated neurotoxicity syndrome (ICANS); fatigue, diarrhea, rash, cough, or endocrine symptoms may be early immune-related adverse events (irAEs). Pediatric assessment is further complicated by developmental variation, limited verbal expression, caregiver–child disagreement, and the effects of prior multimodal therapy. Compared with adult oncology, pediatric surveillance must account for developmental stage, age-dependent communication, caregiver dependence, and lower event frequency, all of which complicate model training, calibration, and bedside interpretation.

Pediatric oncology nurses are therefore not passive recipients of algorithmic alerts. They generate and contextualize much of the information required for safe immunotherapy: repeated vital signs, fluid balance, neurologic observations, pain and behavior assessment, medication reconciliation, family education, and escalation decisions. Artificial intelligence (AI) may improve the integration and prioritization of these signals, but only if the technology is embedded in nursing workflow and retains human oversight. This Mini Review focuses on a nursing-centered model of AI-enabled surveillance that integrates biomarkers, patient- and caregiver-reported outcomes, continuous physiological data, and long-term toxicity management.

### 1.1 Literature search approach

For this Mini Review, we performed a focused literature search in PubMed/MEDLINE, Web of Science, Scopus, and Google Scholar for publications from January 2015 through September 2026, supplemented by earlier landmark trials and guidelines. Search terms combined “pediatric” or “childhood cancer” with “immunotherapy,” “CAR-T,” “bispecific T-cell engager,” “immune checkpoint inhibitor,” “anti-GD2,” “cytokine release syndrome,” “ICANS,” “biomarker,” “nursing,” “patient-reported outcome,” “wearable,” “artificial intelligence,” “machine learning,” and “digital monitoring.”

## 2 Monitoring targets created by pediatric cancer immunotherapy

CAR-T cells and T-cell-engaging antibodies can trigger a systemic inflammatory cascade characterized by immune-cell expansion, cytokine release, endothelial activation, capillary leak, and organ dysfunction. CRS grading is driven by fever, hypotension, and hypoxia, whereas ICANS requires structured neurologic assessment and may include language disturbance, impaired attention, altered consciousness, motor findings, seizures, or cerebral edema (12, 13). Biomarkers such as C-reactive protein, ferritin, interleukin-6, interferon-*γ*, angiopoietin-2, von Willebrand factor, and early cytokine patterns have been associated with severe CRS or neurotoxicity, but none is sufficiently specific to function independently of clinical assessment (14–18). Immune effector cell-associated hemophagocytic lymphohistiocytosis-like syndrome adds a later hyperinflammatory phenotype involving rapidly rising ferritin, cytopenias, coagulopathy, and hepatic injury (19). Pediatric ICANS may be difficult to recognize before overt deterioration, particularly in younger children or those with pre-existing neurodevelopmental differences (20).

Checkpoint inhibitors produce a different surveillance problem. irAEs may affect the skin, gastrointestinal tract, liver, endocrine organs, lungs, heart, kidneys, nervous system, or hematopoietic system and may arise during treatment or after discontinuation. Management guidelines emphasize early recognition, organ-directed evaluation, grading, and timely immunosuppression for clinically significant toxicity (21, 22). Anti-GD2 antibodies can add severe neuropathic pain, hypersensitivity, capillary leak, and autonomic changes. Across modalities, the same observed signal can have competing explanations: tachycardia may reflect pain, dehydration, sepsis, or CRS; new irritability may reflect anxiety, corticosteroids, sleep deprivation, or neurotoxicity. Monitoring must therefore be longitudinal, multimodal, and context aware rather than dependent on a single threshold.

Acute care is followed by a prolonged period of immune and treatment-related vulnerability. Persistent B-cell aplasia, hypogammaglobulinemia, delayed immune reconstitution, recurrent infection, prolonged cytopenia, and late neurologic or psychosocial effects require structured follow-up (23). The clinical target is not merely detection of a severe event after it has occurred, but recognition of a changing trajectory early enough for nursing reassessment and protocol-linked intervention (Table 1).

**Table 1:** Practical surveillance priorities across major pediatric cancer immunotherapy modalities.

Modality	Major toxicities	Candidate biomarkers/digital signals	Nursing assessment	Escalation priority
CAR-T cells/T-cell engagers	CRS, ICANS, infection, cytopenia, IEC-HS	Temperature, BP, SpO2, CRP/ferritin and cytokine trends, neurologic/behavioral change	Frequent vital signs; structured neurologic assessment; ASTCT CRS/ICANS grading; infection screen	Urgent bedside reassessment; sepsis pathway when indicated; clinician escalation; organ support
Immune checkpoint inhibitors	Skin, gastrointestinal, hepatic, endocrine, pulmonary, cardiac, renal, and neurologic irAEs	Organ-specific symptoms, routine organ-function tests, SpO2, ePRO symptom clusters	Focused organ assessment; medication review; longitudinal symptom comparison	Grade- and organ-directed evaluation; urgent assessment for cardiopulmonary/neurologic red flags
Anti-GD2 therapy	Neuropathic pain, hypersensitivity, capillary leak, autonomic changes	Pain score, BP/HR, temperature, oxygenation, fluid balance, behavioral distress	Pain and infusion assessment; perfusion/respiratory monitoring; allergy surveillance	Infusion-related emergency pathway; rapid clinician review; symptom and hemodynamic support

Candidate biomarkers and digital signals are adjunctive. AI-generated risk estimates should prioritize reassessment but must not replace ASTCT CRS/ICANS grading, organ-specific toxicity grading, sepsis evaluation, or bedside clinical judgment. Principles are synthesized from established guidance and nursing literature (12, 13, 21, 22, 24–27).

## 3 Mechanistic rationale for AI-enabled nurse-in-the-loop surveillance

AI is most useful when it converts fragmented observations into a time-dependent estimate of risk. A multimodal model could combine treatment timing, patient-specific baseline values, laboratory trajectories, bedside and wearable vital signs, medication exposures, nursing observations, electronic patient-reported outcomes (ePROs), and caregiver-reported behavioral changes. Time-series methods and anomaly detection are particularly relevant because an individual child's deviation from baseline may be more informative than a population threshold. For example, a small but sustained rise in respiratory rate coupled with declining oxygen saturation, increasing fatigue, and a rising inflammatory marker may warrant review even when no single value is alarming.

The mechanistic value of such a system lies in coupling biological escalation to nursing counter-regulation. Immunotherapy activates cytokine, endothelial, neuroinflammatory, autoimmune, and hematologic pathways; these pathways generate a multimodal digital phenotype. AI can increase temporal sensitivity, identify concordant change across domains, and prioritize patients for reassessment. The nurse then verifies data quality, interprets developmental and clinical context, evaluates competing diagnoses, applies standardized toxicity grading, and initiates escalation. Audited outcomes subsequently inform local threshold adjustment and recalibration (Figure 1). Thus, AI does not replace nursing judgment; it changes the timing and ordering of attention.

**Figure 1:**
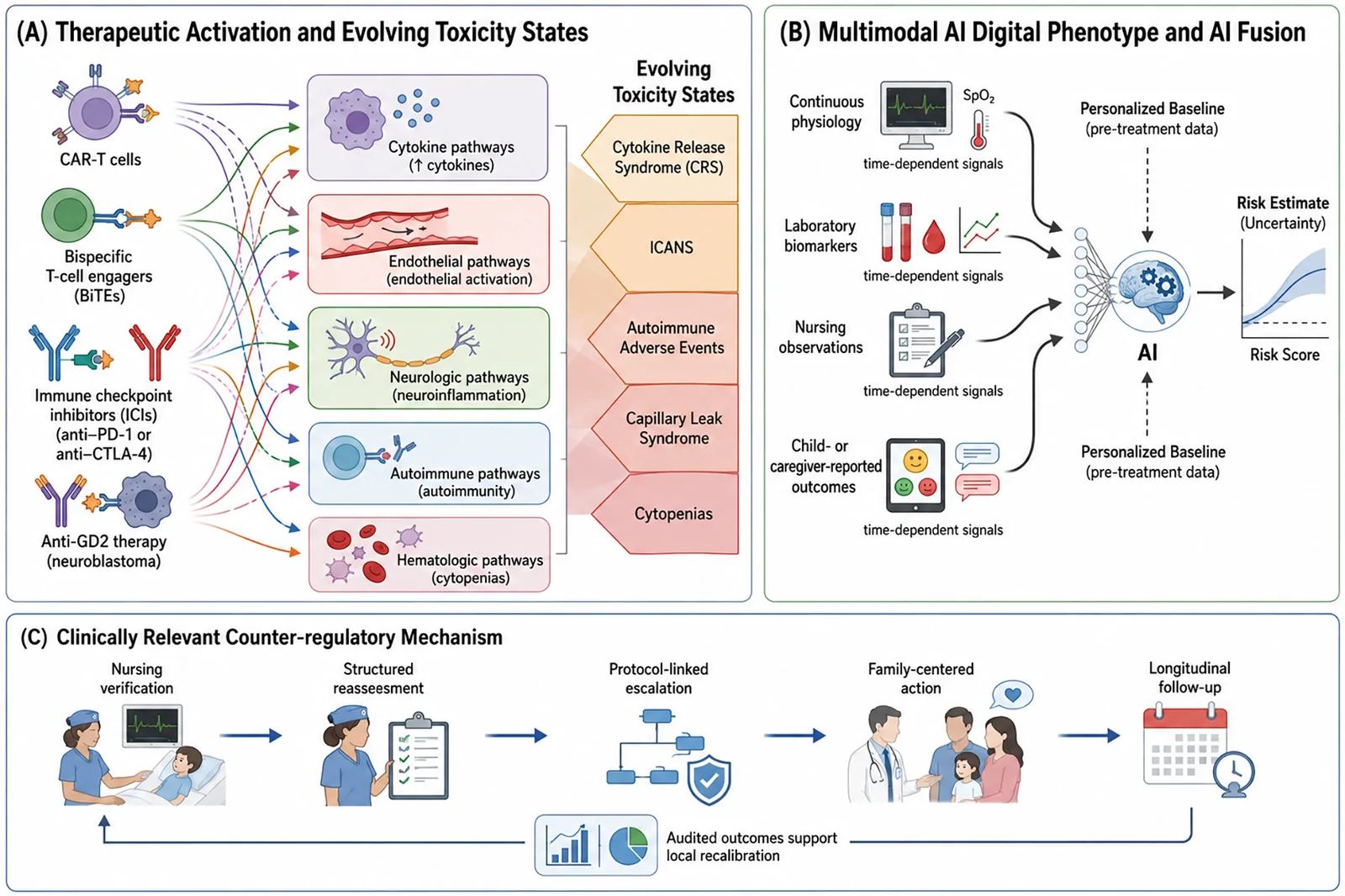
Mechanism of AI-enabled, nurse-in-the-loop surveillance for pediatric cancer immunotherapy. **(A)** CAR-T cells, bispecific T-cell engagers, immune checkpoint inhibitors, and anti-GD2 therapy activate cytokine, endothelial, neurologic, autoimmune, and hematologic pathways that generate evolving toxicity states. **(B)** Continuous physiology, laboratory biomarkers, nursing observations, and child- or caregiver-reported outcomes form a multimodal digital phenotype. AI can fuse these time-dependent signals against a personalized baseline and provide a risk estimate with uncertainty. **(C)** The clinically relevant counter-regulatory mechanism is nursing verification, structured reassessment, protocol-linked escalation, family-centered action, and longitudinal follow-up. AI, artificial intelligence; CAR-T, chimeric antigen receptor T-cell; CRS, cytokine release syndrome; ICANS, immune effector cell-associated neurotoxicity syndrome; ePRO, electronic patient-reported outcome; SpO2, peripheral oxygen saturation.

## 4 Nursing-centered surveillance across the treatment trajectory

Pediatric immunotherapy monitoring should be designed around nursing work rather than added as a parallel technology task. Existing pediatric CAR-T guidance and nursing reviews emphasize preinfusion assessment, standardized observation, family education, rapid recognition of CRS and neurotoxicity, multidisciplinary escalation, infection prevention, and post-discharge follow-up (12, 23–27). AI can strengthen these functions only when the input data, alert route, response responsibility, and documentation pathway are explicit.

### 4.1 Pre-treatment readiness and the personalized baseline

Before treatment, nurses should establish the baseline against which future AI outputs will be interpreted. This includes usual heart rate and blood pressure ranges, oxygen requirement, sleep and activity pattern, hydration status, bowel habits, skin findings, pain phenotype, gait, speech, attention, handwriting or age-appropriate cognitive tasks, and the child's typical response to hospitalization. Baseline laboratory values should be linked to recent transfusion, infection, growth-factor exposure, corticosteroids, organ dysfunction, and prior locoregional or intensive systemic therapy. A model that ignores these modifiers may generate false escalation or falsely reassure.

The nursing assessment should also define the family's monitoring capacity. Caregivers need a clear description of expected symptoms, red flags, who will review electronic reports, how rapidly the team will respond, and when emergency evaluation is required regardless of the AI-generated risk score. Teach-back is essential. Nurses should document language, health literacy, internet and device access, distance from the treatment center, transportation constraints, and whether a caregiver can remain with the child. These variables influence the safety of outpatient monitoring and may be as important as biological risk.

A short baseline ePRO or caregiver-proxy profile can capture fatigue, pain, sleep, anxiety, nausea, bowel symptoms, dyspnea, and functional participation. The purpose is not to create an administrative burden; it is to define the child's normal symptom distribution and the communication channel most likely to be used during deterioration.

### 4.2 Acute bedside monitoring and escalation

During the high-risk period after CAR-T or T-cell engager administration, nurses remain the first continuous interpreters of clinical change. Core observations include temperature, heart rate, blood pressure, respiratory rate, oxygen saturation, perfusion, urine output, fluid balance, weight, pain, mobility, mental status, speech, attention, tremor, seizure activity, and medication exposure. The frequency of assessment should follow institutional protocols and the child's risk trajectory, not the convenience of the algorithm. An AI alert should prompt a structured reassessment rather than automatic treatment. AI-generated risk scores should determine attention priority, not toxicity grade; formal CRS and ICANS grading remains based on ASTCT criteria and bedside findings (13).

Verification begins with signal quality. Nurses determine whether an abnormal wearable value is physiologic or caused by poor contact, movement, anxiety, or device failure. They repeat vital signs, review the timing of antipyretics, sedatives, opioids, transfusions, corticosteroids, and antihypertensives, and examine whether multiple domains are changing together. Infection and CRS may coexist; cultures and antimicrobial pathways should not be delayed because an inflammatory model labels the event as immunotoxicity. Similarly, reduced interaction may reflect fatigue or medication, but a new language or motor abnormality warrants urgent ICANS-focused evaluation.

The response layer should be protocol linked. High-priority alerts must map to a named nursing action: immediate bedside assessment, repeat observations within a defined interval, physician or advanced-practice review, rapid response activation, laboratory testing, cultures, imaging, oxygen and fluid support, or transfer to higher-acuity care. Tocilizumab, corticosteroids, antiseizure therapy, and other treatments remain clinician-prescribed interventions; the nurse's critical contribution is timely recognition, grading, preparation, administration, response monitoring, and documentation. Nursing records should capture not only whether an alert occurred but also why it was accepted, deferred, or overridden. These data are necessary for model audit and for distinguishing useful alerts from noise. When bedside nursing concern is high despite a low predicted risk, clinical assessment should override the algorithm and trigger reassessment or escalation.

Alert fatigue is a direct patient-safety risk. Systems should suppress duplicate notifications, rank alerts by urgency, display the variables driving the score, and show uncertainty. A single unexplained risk number is inadequate. Nurses need to know whether the alert reflects worsening oxygenation, a ferritin trajectory, new caregiver-reported confusion, or missing data. Escalation rules should be tested during simulation, including night shifts and low-staffing periods, before clinical deployment.

### 4.3 Integrating the child's and caregiver's voice

Subjective toxicities are frequently underrecognized when assessment relies only on clinician documentation. The Pediatric Patient-Reported Outcomes version of the Common Terminology Criteria for Adverse Events has demonstrated validity and reliability in children and adolescents, and symptom screening tools such as SSPedi can improve symptom detection and trigger care pathways (28–32). Electronic monitoring is feasible during pediatric cancer hospitalization and in weekly patient- or proxy-reported programs (30, 33). Importantly, reporting is not synonymous with care; symptom data must enter a monitored queue with a defined nursing response.

Age and development determine who reports and how the report should be interpreted. Older children may self-report pain, fatigue, dyspnea, cognitive slowing, and functional interference, whereas younger children may require caregiver proxy reporting and behavioral anchors. Discordance between the child and caregiver is not an error to be averaged away. A child reporting mild symptoms while a caregiver reports marked behavioral change may indicate adaptation, fear of hospitalization, or emerging neurotoxicity. Conversely, caregiver anxiety may amplify perceived severity. Nurses are positioned to reconcile these perspectives through focused questioning and direct observation. Within the proposed AI framework, child and proxy reports should therefore remain distinguishable inputs; the magnitude and direction of discordance can itself be treated as a clinically informative feature or trigger for nursing reassessment rather than mathematically averaged into a single score.

AI-enabled ePRO systems should prioritize change, persistence, clustering, and interference with function rather than simply count symptoms. New combinations—such as poor sleep, headache, slowed responses, and difficulty using a device—may be more informative than any single item. The interface should support accessible language, audio options, proxy switching, and culturally appropriate communication.

### 4.4 Ambulatory and home monitoring

After discharge, the surveillance model changes from continuous bedside observation to intermittent family-generated data. Wearable devices can record multiparameter vital signs in pediatric oncology, demonstrating the technical feasibility of high-frequency physiological monitoring (34). Daily electronic symptom reporting has also been implemented in children with cancer (35). For immunotherapy, the clinically useful home system should combine simple measurements with a nurse-led triage pathway: temperature, oxygen saturation when indicated, activity and sleep trends, oral intake, urine output, pain, diarrhea, rash, cough, neurologic or behavioral change, and medication adherence.

Thresholds must reflect time from therapy, prior toxicity, distance from care, and the reliability of the home data source. A low AI-generated risk score must never prevent families from seeking urgent care. The nursing team should specify response windows—for example, immediate telephone contact for neurologic change or respiratory compromise, same-day review for persistent fever or reduced intake, and scheduled follow-up for low-grade stable symptoms. Systems must also provide a fallback for families without reliable broadband, smartphones, or English proficiency. Digital exclusion should not become a new source of treatment inequity. Neurologic change, respiratory compromise, hemodynamic instability, or persistent fever requires urgent clinical assessment independent of wearable/ePRO output or a low AI-generated risk score.

### 4.5 Long-term toxicity and survivorship nursing

Long-term monitoring must extend beyond the period conventionally associated with CRS or ICANS. Nurses should coordinate surveillance for B-cell aplasia, immunoglobulin deficiency, recurrent infection, delayed vaccination, prolonged cytopenia, fatigue, neurocognitive change, school reintegration, endocrine dysfunction, cardiopulmonary symptoms, growth, puberty, fertility concerns, emotional distress, caregiver burden, and financial toxicity (36–39). Checkpoint inhibitor irAEs may persist or first appear after therapy has ended, while the cumulative effects of prior chemotherapy, radiation, transplantation, and immunotherapy can be difficult to disentangle. CAR-T–specific late concerns include B-cell aplasia, hypogammaglobulinemia, prolonged cytopenia, and infection, whereas checkpoint inhibitors require surveillance for persistent particularly endocrine toxicity. Neurocognitive, cardiopulmonary, growth/pubertal, fertility, psychosocial, and financial effects are broader survivorship concerns that may also reflect prior chemotherapy, radiotherapy, or transplantation. Mechanistic studies outside pediatric immunotherapy demonstrate durable tissue responses to radiation and epigenetic regulation in bone and cardiac models, reinforcing the need to consider prior treatment exposures when interpreting organ-specific late effects (40–42).

AI may support survivorship by assembling longitudinal laboratory and symptom trajectories, identifying missed monitoring, and generating risk-adjusted reminders. It could also flag a pattern that crosses specialties—for example, declining activity, school absence, persistent tachycardia, and endocrine symptoms. The nurse remains responsible for determining whether the signal is clinically meaningful, arranging targeted assessment, coordinating referrals, and explaining the plan to the child and family. Survivorship algorithms should therefore sit within established risk-based follow-up frameworks rather than replace them (43).

### 4.6 Nursing documentation, handover, and quality improvement

AI-enabled monitoring will fail if alerts are detached from nursing communication. Each handover should preserve the treatment day, current risk window, highest prior CRS or ICANS grade, recent biomarker direction, organ-specific symptoms, antimicrobial and immunosuppressive exposure, and the child's developmental baseline. The receiving nurse should be able to see which alerts occurred, what bedside verification found, what action was taken, and when reassessment is due. A closed-loop status—acknowledged, assessed, escalated, resolved, or deferred with rationale—prevents alerts from disappearing into an electronic inbox.

Nursing quality review should examine more than model sensitivity. Useful indicators include time from alert to bedside reassessment, time to clinician escalation, duplicate-alert rate, overnight response, false reassurance, family-initiated urgent contact, unplanned intensive care, and the proportion of alerts without documented closure. Regular case review should include bedside nurses because they can identify data-quality problems and workflow mismatches that are invisible in model performance statistics. These reviews also provide the safest route for adjusting local thresholds: changes should be governed, documented, and reversible rather than silently introduced by software updates.

## 5 Implementation, safety, and research priorities

The evidence base for AI specifically applied to pediatric immunotherapy nursing remains early. Most available studies address general clinical prediction, adult oncology, or feasibility of pediatric digital monitoring rather than prospective improvement in immunotherapy outcomes. Broad claims of automated prediction should therefore be avoided. Machine learning can identify complex patterns, but performance depends on data quality, label definition, sample size, and transportability (44, 45). Pediatric datasets are small, treatment protocols evolve, and severe toxicities are relatively infrequent; models may overfit local practice or fail when transferred to another center. Clinically evaluated pediatric digital approaches currently include ePRO symptom monitoring and wearable feasibility studies, whereas multimodal AI fusion and prospective immunotherapy-specific toxicity forecasting remain largely proposed (30, 33–35).

Implementation should prioritize human factors and safety. Responsible systems require prespecified use cases, transparent input handling, prospective evaluation, error analysis, and explicit description of human–AI interaction (46–48). Nurses should participate in model design, label adjudication, usability testing, and governance because they understand which data are reliable, when documentation lags behind reality, and how alerts alter workload. Outcome measures should include time to bedside reassessment, time to escalation, severe toxicity, unplanned intensive care, symptom burden, family-reported confidence, nursing workload, alert burden, and equity—not only area under the receiver operating characteristic curve.

Bias can arise from unequal device access, missing ePROs, language differences, age-dependent communication, skin-tone effects on sensors, and institutional variation in laboratory timing. A model can appear accurate while systematically underperforming for children who generate less digital data. Algorithmic bias in health care is not theoretical, and subgroup calibration must be reported (49, 50). Privacy and cybersecurity are especially important because pediatric data may include continuous location-linked physiology, family narratives, and long-term developmental information. Consent and assent processes should explain what is collected, who reviews alerts, how long data are retained, and whether data will be used to update models.

A practical research agenda should begin with silent prospective validation, followed by nurse-supervised clinical pilots and multicenter pragmatic evaluation. Models should be recalibrated as products, protocols, and supportive care change. Reporting should follow AI-specific guidance, and clinical governance should require an auditable override mechanism, downtime procedures, and accountability for missed or excessive alerts (47, 48, 51).

## 6 Conclusion

AI-enabled surveillance can strengthen pediatric cancer immunotherapy care by linking biological markers, continuous physiology, nursing observations, and the child's and caregiver's reported experience. Its most plausible clinical mechanism is improved temporal sensitivity: a model identifies a changing multimodal risk state, and a nurse verifies the signal, supplies developmental and treatment context, activates standardized reassessment, and coordinates intervention. The technology should therefore be evaluated as a nurse-in-the-loop safety system, not as an autonomous diagnostic tool.

The nursing contribution spans the full trajectory baseline readiness, acute CRS and ICANS monitoring, irAE recognition, family education, home triage, and long-term survivorship. Future studies must demonstrate that AI improves these nursing processes and patient-centered outcomes without increasing alert fatigue, inequity, or documentation burden. Until such evidence is available, the safest principle is augmentation with accountability: AI may prioritize attention, but pediatric oncology nurses remain the clinical integrators who convert data into timely, family-centered care.
